# Atlantic cod actively avoid CO_2_ and predator odour, even after long-term CO_2_ exposure

**DOI:** 10.1186/1742-9994-10-81

**Published:** 2013-12-27

**Authors:** Fredrik Jutfelt, Maria Hedgärde

**Affiliations:** 1 Department of Biological and Environmental Sciences, University of Gothenburg, PO Box 463, Gothenburg SE-405 30, Sweden; 2 The Sven Lovén Centre for Marine Sciences, Kristineberg 566, Fiskebäckskil SE-451 78, Sweden

**Keywords:** Carbon dioxide, Preference, Teleost, Ocean acidification, Oxygen minimum zone, CO_2_ maximum zone, Olfaction, *Gadus morhua*, GABA, Habituation

## Abstract

**Introduction:**

The rising atmospheric CO_2_ level is continuously driving the dissolution of more CO_2_ into the oceans, and some emission scenarios project that the surface waters may reach 1000 μatm by the end of the century. It is not known if fish can detect moderately elevated CO_2_ levels, and if they avoid areas with high CO_2_. If so, avoidance behaviour to water with high CO_2_ could affect movement patterns and migrations of fish in the future. It is also being increasingly recognized that fish behaviour can be altered by exposure to CO_2_. Therefore this study investigated how long-term exposure to elevated pCO_2_ affects predator avoidance and CO_2_ avoidance in juvenile Atlantic cod (*Gadus morhua*). The fish were exposed to control water or CO_2_-enriched water (1000 μatm) for six weeks before being subjected to tests of behaviour.

**Results:**

Despite long term exposure to elevated pCO_2_ the cod still strongly avoided the smell of a predator. These data are surprising because several coral reef fish have demonstrated reversal of olfactory responses after CO_2_ exposure, turning avoidance of predator cues into preference for predator cues. Fish from both treatment groups also demonstrated strong avoidance of CO_2_ when presented with the choice of control or CO_2_-acidified water, indicating that habituation to the CO_2_ sensory stimuli is negligible.

**Conclusions:**

As Atlantic cod maintained normal behavioural responses to olfactory cues, they may be tolerant to CO_2_-induced behavioural changes. The results also suggest that despite the long-term exposure to CO_2_-acidified water, the fish still preferred the control water over CO_2_-acidified water. Therefore, in the future, fish may alter their movements and migrations in search of waters with a lower CO_2_ content.

## Introduction

Human activities are causing the release of CO_2_ into the atmosphere at increasing rates [1], resulting in a higher oceanic surface partial pressure for CO_2_ (pCO_2_) and a decrease in the pH in a process termed ocean acidification. Currently (may 2013), the levels have reached 400 ppm [2] and could reach 1000 ppm by the year 2100 (the fossil fuel intensive IPCC A1F1 emission scenario [1]), which will result in ~1000 μatm CO_2_ in the surface water [3].

A growing number of reports suggest that the behaviour of coral reef fishes may be highly affected by ocean acidification (see review by Briffa et al. [4]). The behavioural effects appeared at CO_2_ levels predicted by some emission scenarios (700–1200 μatm). A switch from repulsion to attraction to the scent of predators has been observed in several coral reef fish species after CO_2_ exposure. Damselfish larvae became attracted to the smell of predators at 700 μatm, and the larvae completely lost the ability to sense predators at 850 μatm [5]. A similar effect was observed in juvenile coral trout; when the fish were reared in 965 μatm CO_2_, they spent 90% of their time in the predator odour [6]. While the reversing effect of CO_2_-exposure on predator avoidance appears to be common in coral reef fish, it is still unknown if this is ubiquitous in teleosts from other parts of the world.

In order to optimize factors such as temperature, light, food availability and predator density [7], fish navigate through heterogeneous marine environments using many cues [8]. Teleosts employ external chemosensory receptors [2,9], possibly neuroepithelial cells located on the gills [1,10], to detect the ambient CO_2_ concentration. The physiological responses to acute exposure to elevated CO_2_ have been reasonably well described, and include bradycardia, hypertension and hyperventilation [3,11-13]. However, how fish behaviour, distribution and migration might be affected by a heterogeneous CO_2_ environment has received less attention [4,14]. It is known that teleosts avoid water with very high pCO_2_[5,15], and freshwater Arctic charr (*Salvelinus alpinus*) demonstrated attraction to low concentrations of CO_2_ but avoided higher concentrations of CO_2_ in a gradient tank [6,16].

How marine fish behave when presented with the choice of the relatively small concentration gradient of present-day pCO_2_ and a future scenario pCO_2_ is unknown. It is also unknown whether and how long-term exposure and acclimation to elevated CO_2_ levels will influence behavioural choices [7,14,17]. The issue of CO_2_ avoidance behaviour in fish has been highlighted as a concern that experimental biologists should investigate, in a review of possible effects of ocean acidification on fish [8,14]. If marine fish navigate using small differences in pCO_2_, then the increasing pCO_2_ due to anthropogenic disturbance could potentially affect the movement patterns and migrations of fish in the future ocean. For example, areas with heavy macrophyte growth can already reach high CO_2_ levels during the night [18]. With an increased baseline CO_2_ level from ocean acidification, the nightly increase in pCO_2_ in the local microclimate of macrophyte beds could become irritant to some fishes and lead to avoidance of sheltered areas [18].

We investigated whether the ecologically and economically important teleost Atlantic cod actively discriminate between control CO_2_ and elevated CO_2_ levels (1000 μatm), and if this behaviour is modulated by long-term exposure to CO_2_. Furthermore, we tested whether exposure to CO_2_ cause cod to lose the ability to avoid olfactory cues from predators.

## Results

**Figure 1:**
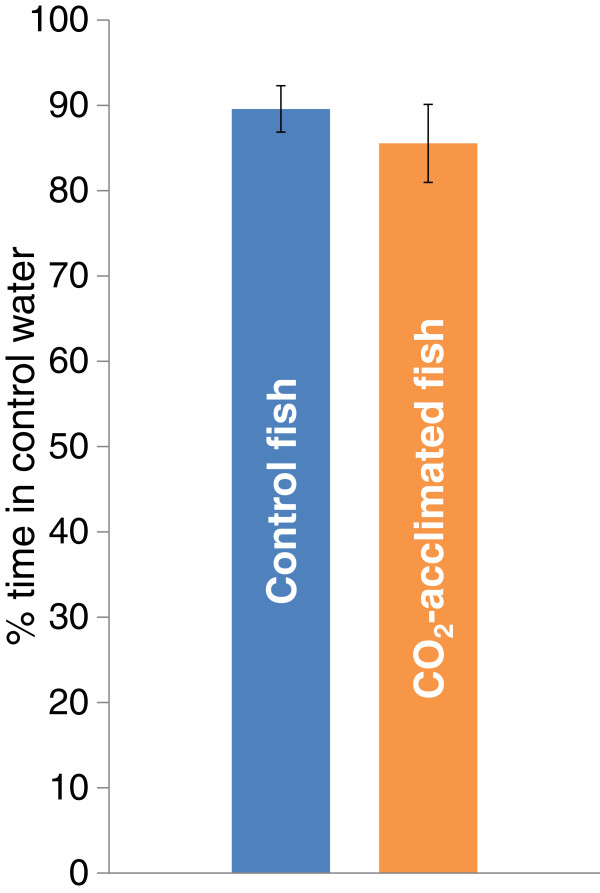
**CO**_**2 **_**avoidance in Atlantic cod after long-term exposure to control water or high pCO**_**2 **_**water.** Mean time (%) that juvenile *Gadus morhua* spent in the control water (550 μatm CO_2_) when presented with the choice of control water or high pCO_2_ water (1170 μatm CO_2_); n_control_ = 19 and n_CO2_ = 12. Fish from both acclimation groups were tested: the control fish and the long-term CO_2_-exposed fish. Both treatment groups displayed equally strong avoidance (p = 0.0001). The data represent the mean ± SEM.

The fish from the control group actively avoided CO_2_-enriched water (p < 0.0001). The CO_2_-exposed fish demonstrated equally strong avoidance behaviour to CO_2_ (p < 0.0001). There was no significant difference between the control fish and CO_2_-exposed fish in the strength of avoidance behaviour (p = 0.482, n_control_ = 19, n_CO2_ = 13) (Figure 1).

**Figure 2:**
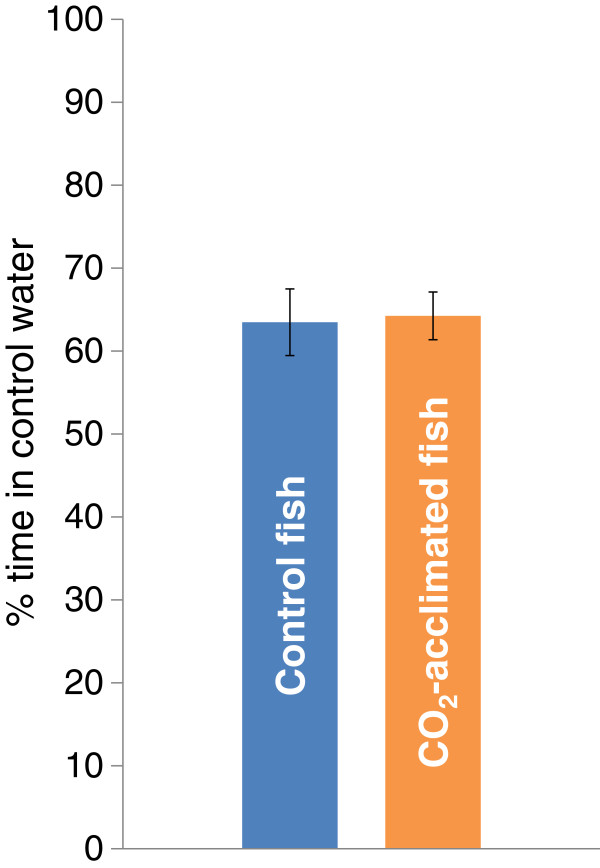
**Predator cue avoidance in Atlantic cod after long-term exposure to control water or high pCO**_**2 **_**water.** Mean time (%) that juvenile *Gadus morhua* spent in the control water, when presented with the choice of control water or water with predator odor; n_control_ = 19 and n_CO2_ = 12. Both treatment groups displayed equally strong avoidance (p = 0.0001). The data represent the mean ± SEM.

In the predator avoidance test (Figure 2), both the control fish and the CO_2_-exposed fish avoided the water coming from the header tank that contained a potential predator (p_Control fish_ < 0.0001 and p_CO2 fish_ <0.0001). There was no significant difference in the strength of the avoidance behaviour between treatments (nested ANOVA; p = 0.481, n_control_ = 18, n_CO2_ = 13).

## Discussion

Increased CO_2_ concentrations in the water can elicit sensory responses in fish [10,13,19] as well as physiological responses [1,12]. However, it has been unclear whether near-future CO_2_-levels can provoke avoidance behaviour in marine fish. In this study, we demonstrated that juvenile Atlantic cod strongly avoided water containing increased pCO_2_ (1000 μatm) in favour of water containing the control CO_2_ concentration (500 μatm). The fish spent 90% of their time in the control water, indicating that elevated pCO_2_ is highly undesirable for young cod.

Surprisingly, the long-term acclimated cod showed the same level of avoidance behaviour as the control fish. This finding suggests that despite the possible acclimation and habituation processes, the cod still considered the high pCO_2_ water as suboptimal and avoided it. Repeated exposure to a sensory stimulus can lead to habituation, and the response to subsequent exposures to the same stimuli has a decreased magnitude [20]. The fish were under constant exposure to high pCO_2_ for over one month, which could theoretically have induced habituation or a shift in the baseline of what is categorized as “normal” for the fish, which would have led to a lower level of avoidance of or possibly even a preference for high CO_2_. Because habituation was not detected, it is possible that the habituation of sensory systems to high CO_2_ is a very slow or non-existent process in fish.

It has been suggested that any CO_2_ avoidance behaviour in fish could affect their distribution, migration patterns and, therefore, the marine ecosystem structure [6,14]. Theoretically, fish could avoid areas of high pCO_2_ and actively seek out lower pCO_2_ waters over both long and short timescales. Daily migrations could be affected in areas with high biomass because the pCO_2_ could already be high because of net respiration during darkness [18,21,22], and combined with ocean acidification, this could drive the CO_2_ levels into the avoidance range for cod.

Pelagic fish normally avoid the mesopelagic oxygen minimum zone, and hypoxia has been suggested as the repellent [7,23,24]. However, these hypoxic zones are also associated with high pCO_2_[8,25,26], which suggests that the avoidance behaviour against oxygen minimum zones observed in nature [9,25] could be associated with high pCO_2_ as well as hypoxia. Because of the close association of hypoxia with hypercapnia in nature, fish may also use hypercapnia as a proxy for harmful oxygen levels despite being tolerant to the CO_2_ itself. While it is too early to draw firm conclusions regarding the ecological relevance of the CO_2_ avoidance behaviour in marine fish, the subject deserves more attention.

Because CO_2_ exposure reverses the preferences to olfactory cues in several coral reef teleost species, as well as affects the behaviour of temperate sticklebacks [27] and gobies [28], we hypothesized that cod would exhibit a reversal of avoidance behaviour. However, cod exposed to CO_2_ for one month avoided both CO_2_ and the predator odour with the same magnitude as the control water-exposed fish, suggesting that the reversal of olfactory preference observed in the tropical reef fish [4] is not ubiquitous among teleost species. Therefore, it is possible that cod is a species that does not demonstrate dramatic behavioural changes following CO_2_ exposure, although this has to be investigated using several independent tests of behaviour. This is corroborated by results from larval Atlantic cod where the larvae maintained normal behaviour despite long term exposure to very high pCO_2_ (4200 μatm) [29]. Neural tolerance to high pCO_2_ in Atlantic cod may be an adapted trait as some populations have been shown to enter hypoxic deep water (≤ 20% O_2_ saturation) to feed [30]. The mechanism for such tolerance is unknown but could involve modulation to the ion permeability of certain neural ion channels, for example the GABA_A_ receptor Cl^-^ channel [31]. As hypoxic deep water is commonly associated with hypercapnia (500–2500 μatm CO_2_ in the Baltic sea [26]), it should be beneficial for cod to maintain normal behaviour despite the high pCO_2_ in the deep water.

## Conclusions

We have shown that Atlantic cod strongly avoided water with elevated CO_2_ levels when given the choice, indicating that cod may navigate using CO_2_ as a cue in a heterogeneous pCO_2_ landscape. The avoidance of high CO_2_ was maintained despite over one month of exposure and acclimation to elevated CO_2_ levels, demonstrating that habituation of the CO_2_ sensory system is minimal. Ocean acidification may therefore alter movement patterns and migrations of fish in the future.

## Materials and methods

### Fish rearing and treatment

The ethical animal experimentation committee (Gothenburg, Sweden, ethical permits Jutfelt 100–2010 and Jutfelt 151–2011) approved the fish handling, exposure and testing.

In total, 56 juvenile Atlantic cod (*G. morhua*) were collected using cages and seine nets close to Sauna Island at the Kristineberg Marine Station, Sweden (lat. 58.2497, long. 11.4455). The fish were measured and weighed at the start and end of the experiment, and randomly distributed in equal numbers to the four tanks. The fish were fed daily with shrimp (*Pandalus* sp.). All mortalities were recorded. At the start of the exposure period the fish had a mean weight of 8.3 g ± 5.0 SD (6.6 g ± 3.7 SD, n = 28, for control fish and 9.9 g ± 5.6 SD, n = 28, for the high pCO_2_ fish); and a mean length of 9.4 cm ± 1.4 SD (9.0 cm ± 1.2 SD for control fish and 9.8 cm ± 1.5 SD for high pCO_2_ fish). At termination of the experiment the control fish had a mean weight of 12.0 g ± 9.3 SD and the high pCO_2_ fish 19.6 g ± 11.8 SD, and the lengths were 10.6 ± 2.2 SD for control fish and 12.5 ± 2.2 SD for high pCO_2_ fish. The total mortality in the CO_2_ tanks was 56%, of which 50% was due to cannibalism and the rest due to unknown causes. The mortality in the control tanks was 28%, of which 29% was due to cannibalism.

Round fiberglass 100 L tanks (4 in total) with flow-through water (taken from 30 meters depth) were used, and each was equipped with a separate 200 L aerated header tank. The fish were exposed to either control water (532 μatm ± 43 SD, which is the normal pCO_2_ for deep water at this location) or water containing elevated CO_2_ (1014 μatm ±76 SD, representing a business as usual emission scenario [1]). The exposure duration was 41 days, representing a sufficient time for growth in the new environment as well as possible acclimation. The tanks were covered with clear polycarbonate sheaths to prevent gas exchange with the atmosphere. The in situ pCO_2_ levels in the fish tanks was measured daily throughout the experiment using an infrared CO_2_ probe (Vaisala GM70, equipped with an aspiration pump, Vaisala, Finland) connected to a thin-walled silicone tubing loop with trapped circulating air in equilibrium with dissolved water gases, as described previously [6,27,32]. The pCO_2_ in the header tanks was maintained using pH-stat computers equipped with pH probes (Aquamedic, Germany), and solenoid valves were used to control the administration of pure CO_2_ (Aga, Sweden). The temperature was recorded continuously, and the mean temperature was 14.4°C ± 0.44 (SD). The alkalinity of the deep water from the Gullmar fjord was measured weekly. The oxygen saturation of the fish tanks was measured on several occasions and was consistently greater than 90% in all tanks. The water carbonate chemistry was calculated using pCO_2_, salinity, temperature, and alkalinity in CO2calc (Hansen, USGS, USA) and the results are presented in Table 1.

**Table 1:** **Water chemistry for the treatments control and elevated CO**_**2**_

**Parameter**	**Control**	**Elevated CO**_**2**_
pCO_2_ (μatm)	532.4 ± 42.7	1013.5 ± 76.0
Alkalinity (TA)	2350 ± 37.1	2363 ± 53.7
Salinity (PSU)	33.1 ± 0.8	33.1 ± 0.8
Temp (°C)	14.4 ± 0.5	14.4 ± 0.5
pH_tot_ (calc.)	7.95 ± 0.04	7.69 ± 0.03
Ωaragonite (calc.)	2.10 ± 0.21	1.22 ± 0.08
Ωcalcite (calc.)	3.29 ± 0.33	1.90 ± 0.13

Temperature, salinity, pCO_2_ and alkalinity (A_T_) are measured data; pH_tot_, Ω_aragonite_ and Ω_calcite_ are calculated data using CO2calc (USGS, USA). Data is presented as means ± SD.

During the exposure period, starting from day 35 and continuing to day 41, the fish were subjected the behavioural tests. Fish from both groups were tested on the same days, ensuring identical exposure and acclimation times for both treatment groups. The fish were always tested in the treatment water pCO_2_, temperature and light conditions they were accustomed.

### Flume choice test

**Figure 3:**
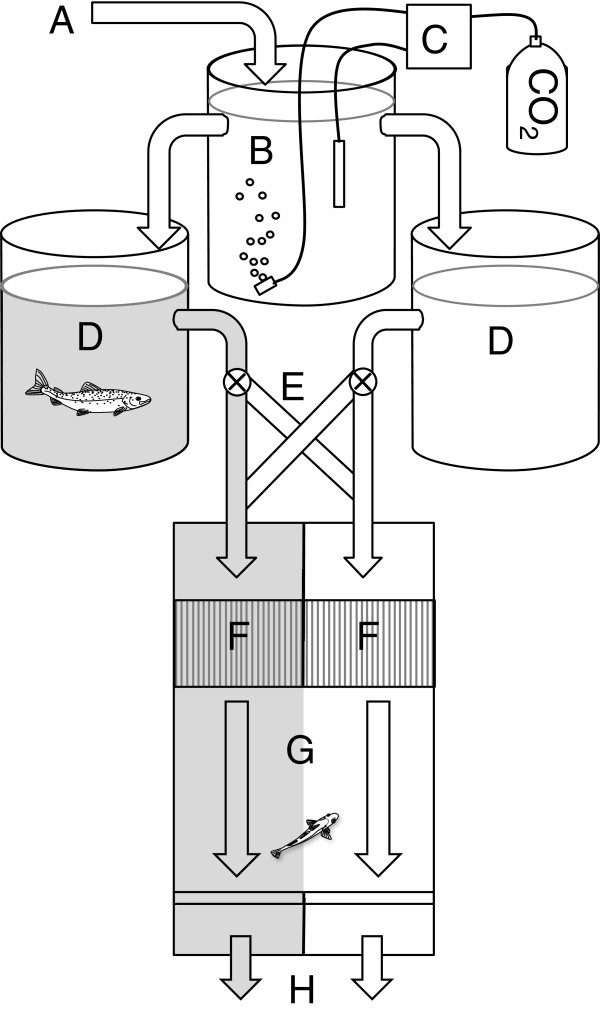
**Choice flume chamber experimental setup with pCO**_**2 **_**control and predator cues on one side.** Choice flume test. Schematic drawing of the choice flume test used for predator avoidance measurements in juvenile cod. The shaded areas represent water with predator odour. The letters represent the following: **A**. Flow-through water inlet. **B**. Main header tank. **C**. pH-stat system with pH probe and solenoid valve controlling the administration of CO_2_ into the main header tank. **D**. Header tanks for the two sides of the choice flume, with one side containing a predatory fish. **E**. Cross-over piping for changing sides of the cue. **F**. Honeycomb plastic for laminar flow. **G**. Choice arena for the tested fish. The two waters were kept separate by laminar flow, and movements only caused minimal short lasting mixing. **H**. Flume drains.

The olfactory flume choice tests and the statistical analyses were performed according to the method described in Gerlach et al. 2007 [33], with some modification to sizes and times described below. A two-choice flume channel (Choice Tank, Loligo Systems, Denmark) containing a 32×40 cm choice arena with a water depth of 15 cm was used to investigate the ability of the cod to detect chemical and olfactory cues (Figure 3). Single fish were placed in the choice arena where the fish could swim freely between two water masses. The two water masses were continuously supplied, in a flow through manner, by two 200 L header tanks, into which cues could be introduced. The two water masses passed through the choice arena with laminar flow at a speed of 1 cm/s, with less than 1 cm of mixed turbulent water between the two laminar flows. The fish could therefore always be determined to be in either of the water masses. The laminar flow of the two parallel water flows was verified by adding dye to one header tank during the method optimization procedure. The pipes connecting the header tanks with the flume were fitted with a crossover switch, which allowed quick change of sides of the olfactory cue without the fish seeing or otherwise noticing the experimenter. A video camera (Dragonfly II, Pointgrey, Richmond, Canada) positioned above the flume was used to record each trial. Individual fish were placed in the flume (marked “G” in Figure 3 and filmed for 20 minutes before the flows were switched, and the olfactory cue switched side of the flume within one minute. After the switch, the flow was maintained for 12 minutes. The time before the switch was longer because the fish needed time to calm down after handling before responding to olfactory cues (according to our method development for this species and life stage). Analysis of fish positions was performed during the last 5 minutes prior to the switch and the last 5 minutes after the switch. The position of the fish was recorded every five seconds. The preferred side for each fish was calculated as the side that the fish spent more than 50% of the time in during each 5-minute period. Each fish was then used as its own control in the non-parametric statistical test; the preferred side before the switch was compared to the preferred side after the switch, see the statistics section below for details. A lack of preference (50% of the time in each water mass) would be expected if the fish did not prefer one olfactory cue, or if the fish were unable to detect the cue.

### Predator avoidance

Predator avoidance was tested at 40 and 41 days of exposure. The flume choice test described above was used to investigate the ability of the cod to detect a predator cue (Figure 3) [34]. Brown trout (*Salmo trutta*) is a piscivore that inhabits the same biotope as juvenile cod [35]; therefore, this fish is a likely predator of small cod. A main header tank (200 L) (marked “B” in Figure 3) with aeration and pCO_2_ control was used to supply the two downstream header tanks (200 L) (marked “D” in Figure 3), making it possible to use the pCO_2_ that the fish were acclimated to on both sides of the flume simultaneously, so that the CO_2_ concentration did not affect the choice of water mass. The two header tanks with flow-through water (one control tank and one tank containing a live wild-caught 500 g trout) supplied water to the two sides of the flume at a flow rate of 17 L/minute each.

### CO_2_ avoidance

CO_2_ avoidance was tested at 35 to 37 days of exposure. The two-choice flume channel used to investigate the ability of cod to detect elevated CO_2_ levels (1000 μatm) was similar to the setup for predator avoidance but without header tank “B” Figure 3. Two header tanks with flow-through water (tanks “D” in Figure 3 one control tank (500 μatm) and one tank with an elevated CO_2_ concentration (1000 μatm)) supplied the two sides of the flume at a flow rate of 17 L/minute each. The same protocol for video analysis as in the predator avoidance test was used.

### Data analysis

Nested ANOVAs (tank nested within treatment) in SPSS were used to investigate treatment (the long-term exposure to control water or elevated pCO_2_) and tank effects on CO_2_ avoidance and predator avoidance. The ANOVAs used the % of time spent in control water as the variable. No tank effects were detected for any parameter (at the p < 0.1 level). The non-parametric Wilcoxon signed-rank test was used to detect possible CO_2_ and predator avoidance within treatments, in which the position (in control water mass or in water mass with olfactory cues) of the individual fish was compared before and after the switching of the side with odour or CO_2_. This method of statistically testing preference and avoidance is described in Gerlach et al. 2007. The data are presented as the mean ± SEM, unless otherwise noted.

## Competing interests

The authors declare that they have no competing interests.

## Authors’ contributions

FJ and MH designed and conducted the experiments. MH performed the statistical analysis. FJ wrote the manuscript. Both authors read and approved the final manuscript.

## Authors’ information

FJ and MH address: Department Biological and Environmental Sciences, University of Gothenburg, Medicinaregatan 18, 41390 Gothenburg, Sweden. Secondary address: The Sven Lovin Centre Kristineberg, Kristineberg 566, SE-451 78 Fiskebäckskil, Sweden.

## References

[B1] Solomon S Qin D Manning M Alley RB Berntsen T Wratt D Contribution of Working Group I to the Fourth Assessment Report of the Intergovernmental Panel on Climate Change 2007 Cambridge, UK and NY, USA: Cambridge University Press

[B2] National Oceanic and Atmospheric Administration, Earth System Research Laboratory http://www.esrl.noaa.gov/news/2013/CO2400.html

[B3] Doney SC Ruckelshaus M Emmett Duffy J, Barry JP, Chan F, English CA, Galindo HM, Grebmeier JM, Hollowed AB, Knowlton N, Polovina J, Rabalais NN, Sydeman WJ, Talley LD: **Climate Change Impacts on Marine Ecosystems** Annu Rev Marine Sci 2012 10 11 37 10.1146/annurev-marine-041911-111611 22457967

[B4] Briffa M La Haye De K Munday PL **High CO2 and marine animal behaviour: potential mechanisms and ecological consequences** Mari Pollut Bull 2012 10 1519 1528 10.1016/j.marpolbul.2012.05.032 22749063

[B5] Munday PL Dixson DL McCormick MI Meekan M Ferrari MCO Chivers DP Replenishment of fish populations is threatened by ocean acidification Proc Natl Acad Sci USA 2010 10 12930 12934 10.1073/pnas.1004519107 20615968 PMC2919925

[B6] Munday PL Pratchett MS Dixson DL Donelson JM Endo GGK Elevated CO2 affects the behavior of an ecologically and economically important coral reef fish Mar Biol 2012 10 2137 2144 Available: http://www.springerlink.com/index/10.1007/s00227-012-2111-6

[B7] Righton DA Andersen KH Neat F Thorsteinsson V Steingrund P Svedäng H Michalsen K Hinrichsen HH Bendall V Neuenfeldt S Wright P Jonsson P Huse G van der Kooij J Mosegaard H Hüssy K Metcalfe J Thermal niche of Atlantic cod Gadus morhua: limits, tolerance and optima Mar Ecol Prog Ser 2010 10 1 13

[B8] Hara TJ SMELL, TASTE, AND CHEMICAL SENSING | Chemoreception (Smell and Taste) Farrell AP An Introduction Encyclopedia of Fish Physiology 2011 San Diego: Academic Press 183 186 ISBN 9780080923239, http://dx.doi.org/10.1016/B978-0-12-374553-8.00021-6. Keywords: Gustation; Olfaction; Smell; Taste

[B9] Perry SF Gilmour KM Sensing and transfer of respiratory gases at the fish gill J Exp Zool 2002 10 249 263 10.1002/jez.10129 12115900

[B10] Qin Z Lewis JE Perry SF Zebrafish (Danio rerio) gill neuroepithelial cells are sensitive chemoreceptors for environmental CO2 J Physiol 2010 10 861 872 10.1113/jphysiol.2009.184739 20051495 PMC2834944

[B11] Hara TJ The diversity of chemical stimulation in fish olfaction and gustation Rev Fish Biol Fisheries 1994 10 1 35 10.1007/BF00043259

[B12] Perry SF Abdallah S Mechanisms and consequences of carbon dioxide sensing in fish Respir Physiol Neurobiol 2012 10 309 315 10.1016/j.resp.2012.06.013 22705499

[B13] Yoshii K Kashiwayanagi M Kurihara K Kobatake Y High sensitivity of the eel palatine receptors to carbon dioxide Comp Biochem Physiol A Comp Physiol 1980 10 327 330 10.1016/0300-9629(80)90170-X

[B14] Ishimatsu A Physiological effects on fishes in a high-CO 2world J Geophys Res 2005 10 C09S09

[B15] Clingerman J Bebak J Mazik PM Summerfelt ST Use of avoidance response by rainbow trout to carbon dioxide for fish self-transfer between tanks Aquacultural Eng 2007 10 234 251 10.1016/j.aquaeng.2007.07.001

[B16] Jones KA Hara TJ Scherer E Locomotor response by Arctic char (Salvelinus alpinus) to gradients of H+ and CO2 Physiological zoology 1985 10 413 420

[B17] Ishimatsu A Hayashi M Kikkawa T Fishes in high-CO2, acidified oceans Mar Ecol Prog Ser 2008 10 295 302

[B18] Saderne V Fietzek P Herman PMJ Extreme variations of pCO2 and pH in a macrophyte meadow of the baltic sea in summer: evidence of the effect of photosynthesis and local upwelling PLoS One 2013 10 e62689 10.1371/journal.pone.0062689 23626849 PMC3633870

[B19] Hara TJ Olfaction and gustation in fish: an overview Acta Physiol Scand 1994 10 207 217 10.1111/j.1748-1716.1994.tb09800.x 7839864

[B20] Maximino C de Brito TM da Silva Batista AW Herculano AM Morato S Gouveia A Jr Measuring anxiety in zebrafish: a critical review Behav Brain Res 2010 10 157 171 10.1016/j.bbr.2010.05.031 20510300

[B21] Cripps IL Munday PL McCormick MI Ocean acidification affects prey detection by a predatory reef fish PLoS One 2011 10 e22736 10.1371/journal.pone.0022736 21829497 PMC3145675

[B22] Hofmann GE Barry JP Edmunds PJ Gates RD Hutchins DA Klinger T Sewell MA The effect of ocean acidification on calcifying organisms in marine ecosystems: an organism-to-ecosystem perspective Annu Rev Ecol Evol Syst 2010 10 127 147 10.1146/annurev.ecolsys.110308.120227

[B23] Stramma L Prince ED Schmidtko S Luo J Hoolihan JP Visbeck M Wallace DWR Brandt P Körtzinger A Expansion of oxygen minimum zones may reduce available habitat for tropical pelagic fishes Nat Climate Change 2011 10 33 37 10.1038/nclimate1304

[B24] Seibel BA Critical oxygen levels and metabolic suppression in oceanic oxygen minimum zones J Exp Biol 2010 10 326 336 10.1242/jeb.049171 21177952

[B25] Paulmier A Ruiz-Pino D Garçon V CO_2_ maximum in the oxygen minimum zone (OMZ) Biogeosciences 2011 10 239 252 10.5194/bg-8-239-2011

[B26] Melzner F Thomsen J Koeve W Oschlies A Gutowska MA Future ocean acidification will be amplified by hypoxia in coastal habitats Mar Biol 2012 10 1875 1888 doi:10.1007/s00227-012-1954-1

[B27] Jutfelt F Bresolin de Souza K Vuylsteke A Sturve J Behavioural disturbances in a temperate fish exposed to sustained high-CO2 levels PLoS ONE 2013 10 e65825 doi:10.1371/journal.pone.0065825 10.1371/journal.pone.0065825 23750274 PMC3672104

[B28] Forsgren E Dupont S Jutfelt F Amundsen T Elevated CO2 affects embryonic development and larval phototaxis in a temperate marine fish Ecol Evol 2013 10 3637 3646 doi:10.1002/ece3.709 10.1002/ece3.709 24198929 PMC3810864

[B29] Maneja RH Frommel AY Browman HI Clemmesen C Geffen AJ The swimming kinematics of larval Atlantic cod, Gadus morhua L., are resilient to elevated seawater pCO2 Mar Biol 2012 10 1963 1972 doi:10.1007/s00227-012-2054-y

[B30] Neuenfeldt S Andersen KH Hinrichsen HH Some Atlantic cod Gadus morhuain the Baltic Sea visit hypoxic water briefly but often J Fish Biol 2009 10 290 294 10.1111/j.1095-8649.2009.02281.x 20738498

[B31] Nilsson GE Dixson DL Domenici P McCormick MI Sørensen C Watson S-A Munday PL Near-future carbon dioxide levels alter fish behaviour by interfering with neurotransmitter function Nat Climate Change 2012 10 201 204 10.1038/nclimate1352

[B32] Hari P Pumpanen J Huotari J Kolari P Grace J Vesala T Ojala A High-frequency measurements of productivity of planktonic algae using rugged nondispersive infrared carbon dioxide probes Limnol Oceanogr Methods 2008 10 347 354

[B33] Gerlach G Atema J Kingsford MJ Black KP Miller-Sims V Smelling home can prevent dispersal of reef fish larvae Proc Natl Acad Sci USA 2007 10 858 863 10.1073/pnas.0606777104 17213323 PMC1783404

[B34] Herbert NA Skjæraasen JE Nilsen T Salvanes AGV Steffensen JF The hypoxia avoidance behaviour of juvenile Atlantic cod (Gadus morhua L.) depends on the provision and pressure level of an O2 refuge Mar Biol 2010 10 737 746

[B35] Wennhage H Pihl L Fish feeding guilds in shallow rocky and soft bottom areas on the Swedish west coast J Fish Biol 2002 10 sa 207 228 10.1111/j.1095-8649.2002.tb01772.x

